# Expression of FcRL4 defines a pro-inflammatory, RANKL-producing B cell subset in rheumatoid arthritis

**DOI:** 10.1136/annrheumdis-2013-204116

**Published:** 2014-01-15

**Authors:** L Yeo, H Lom, M Juarez, M Snow, C D Buckley, A Filer, K Raza, D Scheel-Toellner

**Affiliations:** 1 Rheumatology Research Group, Centre for Translational Inflammation Research, College of Medical and Dental Sciences, University of Birmingham, Birmingham, UK; 2 University Hospitals Birmingham NHS Foundation Trust, Birmingham, UK; 3 Royal Orthopaedic Hospital NHS Foundation Trust, Birmingham, UK; 4 Sandwell and West Birmingham Hospitals NHS Trust, Birmingham, UK

**Keywords:** Cytokines, Rheumatoid Arthritis, Synovitis, Inflammation, B cells

## Abstract

**Objectives:**

The success of B cell targeting therapies has highlighted the importance of B cells in rheumatoid arthritis pathogenesis. We have previously shown that B cells in the RA synovium are capable of producing pro-inflammatory and bone-destructive cytokines including RANKL. Here we sought to characterise the nature and functional relevance of the RANKL-producing B cell subset in the RA synovium.

**Methods:**

Synovial fluid and peripheral blood B cells from patients with RA were analysed by flow cytometry for markers of B cell differentiation and activation and for chemokine receptors. FcRL4^+^ and FcRL4^−^ B cells sorted from synovial fluid were analysed for cytokine expression using Taqman low-density arrays. Synovial tissue biopsies obtained from patients with RA were analysed by immunofluorescence for CD20, RANKL and FcRL4. FCRL4 mRNA expression was determined in synovial tissue of RA patients and non-inflammatory control subjects by real-time PCR.

**Results:**

RANKL-producing B cells in RA synovial tissue and fluid were identified as belonging to a distinct subset of B cells defined by expression of the transmembrane protein FcRL4. FcRL4+ B cells express a distinct combination of cytokines and surface proteins indicating a function distinct from that of FcRL4− B cells. Notably, FcRL4+ B cells expressed high levels of TNF-α and RANKL mRNA.

**Conclusions:**

We have identified a novel pro-inflammatory B cell population in the RA synovium which is defined by expression of FcRL4 and responsible for RANKL production. This B cell population expresses high levels of CD20, and its removal by rituximab may contribute to the anti-inflammatory effect of this drug.

## Introduction

The effectiveness of B cell depleting anti-CD20 antibodies such as rituximab in reducing synovial inflammation and progression of structural damage in RA suggests B cells are critically involved in these processes.^1^ ^2^ Potential pathogenic attributes of B cells include their ability to produce autoantibodies, act as antigen presenting cells, and secrete cytokines.^3^ We have recently described the production of a range of cytokines including RANKL, TNF-α and IL-6 by synovial B cells.^4^ RANKL plays a critical role in stimulating the differentiation and activation of bone-resorbing osteoclasts, while pro-inflammatory cytokines such as TNF-α and IL-6 contribute to erosion by stimulating production of RANKL, directly acting on osteoclasts and their precursors, and stimulating matrix metalloproteinase production by other cell types.^5–8^ RANKL also has an important role in lymphoid development with RANKL-deficient mice lacking all lymph nodes.^7^ Levels of RANKL in the synovium are significantly reduced following treatment with rituximab, suggesting a link between B cells, inflammation and joint destruction.^9^

RANKL expression has been reported in a FcRL4 positive subset of memory B cells present in the human tonsil.^10^ Fc-receptor-like-4 (FcRL4) is a transmembrane protein with homology to Fc receptors which is capable of aborting B cell receptor-mediating signalling and proliferation, and may therefore play an important role in the regulation of B cell activation and differentiation.^11^ ^12^ Expression of FcRL4 is restricted to B cells and across 950 cancer cell lines is only detected in B cell tumour lines (EBI Gene Expression Atlas). FcRL4 is expressed predominantly in the tonsil, with lower levels detected in the salivary gland, spleen and tongue (EBI Gene Expression Atlas, NCBI GeoProfiles database). In the present study, we have identified a subset of B cells expressing FcRL4 in the rheumatoid synovium. This FcRL4+ B cell population is capable of producing the bone-destructive and pro-inflammatory cytokines RANKL and TNF-α, and is thus likely to represent a pathogenic B cell subset.

## Methods

### Patients

Synovial fluid and peripheral blood were obtained from patients with long-standing RA fulfilling 1987 American College of Rheumatology (ACR) classification criteria.^13^ Synovial tissue was obtained from 25 newly presenting patients with rheumatoid arthritis (RA) or undifferentiated arthritis (UA) that evolved into RA during follow-up, and eight control subjects undergoing arthroscopy for assessment of non-inflammatory conditions. Patient details are provided in table 1. The study was conducted in compliance with the Helsinki declaration, and ethical approval was obtained from the local ethics committee. All subjects gave informed, written consent.

### Flow cytometry and cell sorting

Synovial fluid was incubated with 1000 U/mL endotoxin-free hyaluronidase (Wockhardt UK) at 37°C for 15 min. Mononuclear cells were isolated from synovial fluid and peripheral blood using density gradient centrifugation. Cells were stained with mouse monoclonal antibodies against CD19 (Biolegend), CD20 (Invitrogen), CD27 (BD Pharmingen), IgD (eBioscience), CD11c (Biolegend), RANKL (eBioscience), FcRL4 (Biolegend), CD95 (BD Pharmingen), CD21 (eBioscience), CD80 (BD Biosciences), CD86 (Biolegend), CCR1 (R&D Systems) and CCR5 (BD Biosciences). Isotype, concentration, species and label-matched control antibodies were used. Data were acquired using a Dako Cyan ADP flow cytometer and analysed using SUMMIT software. Synovial fluid mononuclear cells were stained with antibodies against CD19 (Immunotools) and FcRL4 (Biolegend) and sorted using a MoFlo cell sorter (Dako). Sorted populations had a purity of >95%. Mononuclear cells were also isolated from mechanically dissociated synovial tissue as previously described^14^ from RA patients undergoing joint-replacement surgery which were assessed by flow cytometry for FcRL4 and CD19[Table ANNRHEUMDIS2013204116TB1].

**Table 1 ANNRHEUMDIS2013204116TB1:** Clinical characteristics of patients

	RA patients from whom synovial fluid was obtained	Newly presenting patients with RA or UA that evolved into RA during follow-up from whom synovial tissue was obtained
Patients, n	23	25
Female, n (%)	15 (65)	15 (60)
Age yrs; median (IQR)	55 (44–68)	58 (47–65)
Disease duration; median (IQR)	7 years (1–11)	12 weeks (6–38)
RF-positive, n (%)	13 (57)	12 (48)
CCP-positive, n (%)	10 (53)*	14 (56)
CRP, mg/mL; median (IQR)	20 (7–45)	18 (8–42)
ESR, mm/h; median (IQR)	36 (20–43)	27 (16–57)
SJC (28); median (IQR)	4 (2–9)	7 (4–15)
TJC (28); median (IQR)	5 (2–10)	7 (3–15)
DAS28 ESR; median (IQR)	5.2 (3.6–5.6)	5.2 (4.3–6.5)
DMARDs, n receiving (%)	16 (70)	1 (4)

*CCP information was available for 19/23 patients.

CCP, cyclic citrullinated peptide; CRP, C-reactive protein; ESR, erythrocyte sedimentation rate; SJC, swollen joint count; TJC, tender joint count.

### Immunofluorescence

Staining was performed on 5 µm frozen tissue sections. Mouse anti-CD20 (Dako) was developed with goat anti-mouse IgG2a FITC (Southern Biotech), rabbit anti-RANKL (AbCam) was developed with donkey anti-rabbit Rhodamine (Jackson ImmunoResearch), and mouse anti-FcRL4 (BioLegend) was developed with goat anti-mouse IgG2b Cy5 (Southern Biotech). Isotype-matched irrelevant controls were used (Jackson ImmunoResearch). Sections were incubated with primary antibody for 1 h and secondary antibody for 30 min. Sections were mounted using a DAPI-containing mounting medium. Sections were visualised using a Zeiss confocal LSM 510 microscope and images were processed using Zeiss LSM Image Examiner software.

### Immunohistochemistry

Staining was performed on 5 µm frozen acetone-fixed sections. Endogenous peroxidase activity was blocked with Bloxall Solution (Vector) and 2% casein was used to block non-specific staining. Sections were washed with phosphate buffered saline (PBS) between incubation steps. Sections were incubated with anti-CD20 (Dako), mouse anti-FcRL4 (Biolegend), or isotype-matched irrelevant controls (Jackson ImmunoResearch) for 1 h, rabbit anti-mouse Ig (Dako) for 10 min, and anti-rabbit Ig peroxidase (Vector Laboratories) for 30 min. Staining was developed using a diaminobenzidine substrate (Vector Laboratories). Slides were counterstained with haematoxylin and mounted with dibutyl phthalate xylene (DPX) (Sigma Aldrich).

### Real-time PCR

TaqMan low-density real-time PCR arrays (Applied Biosystems) were designed to determine expression of the following genes: IL-1α, IL-1β, IL-2, IL-4, IL-5, IL-6, IL-7, IL-10, IL-11, IL-12p35, IL-12p40, IL-13, IL-15, IL-17A, IL-18, IL-21, IL-22, IL-23p19, IL-27, TNF-α, LT-β, RANKL, APRIL, BAFF, TGF-β1, CSF-2, CSF-3, MIF, EGF, FGF2, VEGF-α, IFN-γ, IFN-α1, IFN-β, CCL2, CCL3, CCL4, CCL5, CCL11, CXCL8, CXCL12, GAPDH and 18S. RNA was extracted from sorted B cells (minimum 10^3^ cells per sample) using a Nucleospin XS kit (Machery-Nagel). A reaction mixture containing 49 µL RNA, 50 µL Quantitect-RT Master Mix (Qiagen) and 1 μL Quantitect Reverse Transcriptase (Qiagen) was added to a TaqMan microfluidic card. Reverse transcription and real-time PCR was performed in a 7900HT Real-Time PCR System (Applied Biosystems). The cycling program used was 50°C for 30 min, 94.5°C for 15 min, then 40 cycles of 96°C for 30 s and 59.7°C for 1 min. FcRL4 expression was determined in synovial tissue from RA patients and non-inflammatory controls. RNA was extracted from 20 μm synovial tissue sections using the RNeasy kit (Qiagen), reverse transcribed using the Superscript VILO kit (Invitrogen), then used in real-time PCR assays containing 12.5 μL Taqman Master Mix, 1 μL FcRL4 or 18S assay (all Applied Biosystems), 6.5 μL H_2_O and 5 μL cDNA. The cycling programme used was 50°C for 2 min, 95°C for 10 min and 40 cycles of 15 s at 95 and 1 min at 60°C. Relative gene expression (RQ) was expressed as 2^−Δ^^C^^t^.

### Statistical analysis

The Wilcoxon-matched paired test was used to assess differences in matched samples or cell populations. The Mann–Whitney test was used to assess differences between two groups. The Kruskal–Wallis test and Dunn's post-test were used to compare >2 groups. The Spearman correlation test was used to assess correlation. Medians are shown on dot plots. All statistical analyses were carried out using GraphPad Prism software.

## Results

### Identification of FcRL4+ B cells in the rheumatoid synovium

**Figure 1:**
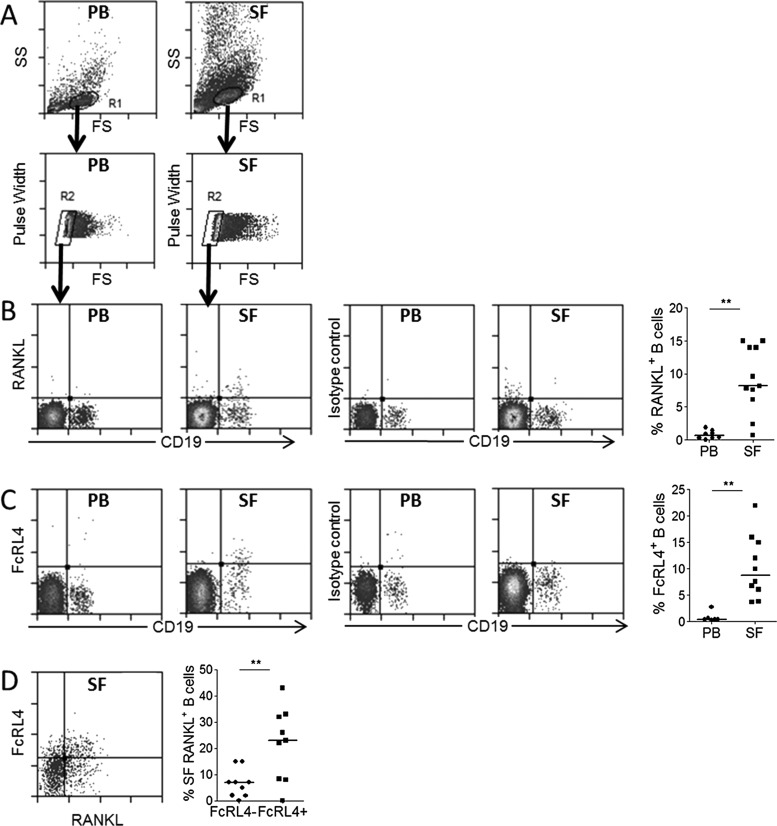
Identification of FcRL4+ B cells in the RA synovial fluid (SF). (A) Cells were gated on a forward and side scatter pattern typical for lymphocytes (R1). Doublets were excluded based on pulse width versus forward scatter properties (R2). (B) Flow cytometric analysis of mononuclear cells from peripheral blood (PB) and SF shows RANKL expression by B cells is significantly higher in SF compared with PB. (C) FcRL4 is expressed by B cells in SF at a significantly higher frequency than PB. (D) RANKL is expressed by FcRL4+ B cells in SF, with RANKL+ B cells significantly enriched in the FcRL4+ B cell population. **p<0.01, Wilcoxon paired test.

Analysis of matched synovial fluid and peripheral blood mononuclear cells from established RA patients by flow cytometry showed that RANKL-producing B cells are present in the synovial fluid, and are significantly enriched in this compartment compared to peripheral blood (figure 1A,B). RANKL has previously been detected in tonsillar memory B cells defined by surface expression of the FcRL4 receptor. We therefore sought to determine whether RANKL+ B cells present in RA synovial fluid belonged to the FcRL4+ B cell population. Synovial fluid and peripheral blood mononuclear cells were stained for FcRL4 and RANKL in addition to the B cell marker CD19. In the synovial fluid, FcRL4 was found to be expressed by a subset of B cells (comprising a median of 13% of B cells), and this population was found at a significantly higher frequency in synovial fluid compared to peripheral blood (median 0.4%) (figure 1C). The percentage of B cells among the synovial fluid mononuclear cells ranged from 1.1% to 6.3% (median 1.3%, n=10, data not shown). Assessment of coexpression of FcRL4 and RANKL showed that RANKL was indeed expressed by FcLR4+ B cells, with RANKL+ B cells found to be significantly enriched in the FcRL4+ B cell population compared to the FcRL4− B cell fraction (figure 1D). The variability in RANKL expression by FcRL4+ B cells may reflect the requirement of FcRL4+ B cells to receive a stimulatory signal in order to express RANKL. No significant correlation was observed between the percentage of FcRL4+ and/or RANKL+ B cells in synovial fluid and disease activity (ESR or DAS28 ESR).

**Figure 2:**
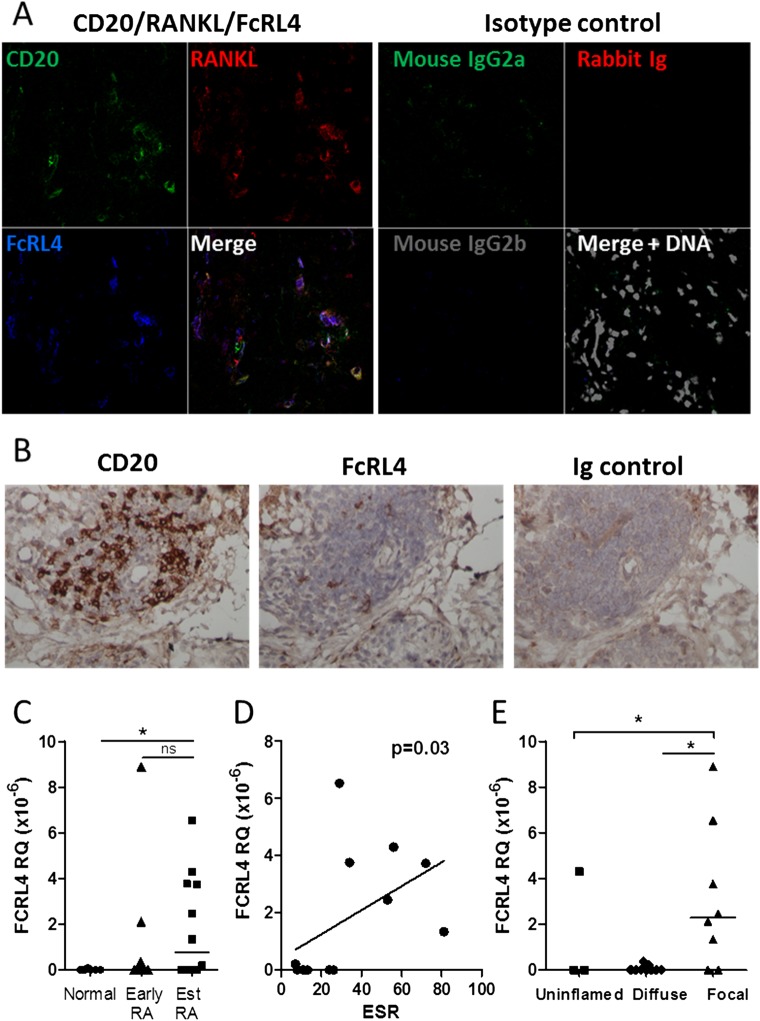
FcRL4 is present in the RA synovium. (A) Established RA synovial tissue stained for CD20 (green), FcRL4 (blue) and RANKL (red) using immunofluorescence. Coexpression of FcRL4 and RANKL is detected in CD20+ B cells. Image representative of n=5. (B) Localisation of FcRL4+ CD20+ B cells in established RA synovial tissue by immunohistochemistry. Representative of n=3. (C) FCRL4 mRNA expression in synovium from non-inflamed controls (n=8), early RA patients (n=13) and established RA patients (n=12). FcRL4 expression is significantly higher in RA synovium. *p<0.05, Mann–Whitney test. (D) Correlation between FcRL4 expression and ESR in established RA patients. (E) FcRL4 expression in synovial tissue from newly presenting RA, UA that evolved into RA during follow-up, or established RA, is highest in biopsies containing focal lymphocyte aggregates. *p<0.05, Kruskal–Wallis test (overarching bar) and Dunn's post-test (underlying bar).

In order to determine if FcRL4+ B cells were present in the inflamed RA synovium itself, synovial tissue sections from patients with established RA were stained for FcRL4, RANKL and CD20. FcRL4− expressing B cells were detected in synovial tissue, and these were found to express RANKL (figure 2A). FcRL4+ B cells were predominantly localised beneath the synovial lining layer and around blood vessels (figure 2B). Additionally, FcRL4 mRNA expression was assessed in synovial tissue from established RA patients and non-inflammatory control subjects. mRNA expression of FcRL4 was significantly elevated in established RA patients compared to non-inflammatory controls (figure 2C). FcRL4 mRNA expression was also upregulated in a proportion of early RA patients from whom samples were obtained within the first 3 months of symptom onset (figure 2C). This indicated that FcRL4+ B cells are not present in normal synovium but develop or accumulate in RA. FcRL4 mRNA expression in the synovium of RA patients significantly correlated with ESR (p=0.03, R=0.64, figure 2D), suggesting an association between the presence of FcRL4+ B cells and the degree of inflammation. The type of leukocyte infiltrate in synovial tissue samples from newly presenting patients with RA or UA that evolved into RA during follow-up was graded as uninflamed, diffuse or focal (containing lymphocyte aggregates) by examining H&E-stained biopsies. FcRL4 mRNA expression in matched biopsies was significantly increased in samples with focal aggregates compared to those with diffuse leukocyte infiltration (figure 2E). We used flow cytometry to assess FcRL4 expression in mechanically dissociated synovial tissue from established RA patients. A mean of 16% of B cells (range 8–32%) was recovered from synovial tissue expressed FcRL4 (n=4, data not shown).

### Phenotypic characterisation of synovial fluid FcRL4+ B cells

**Figure 3:**
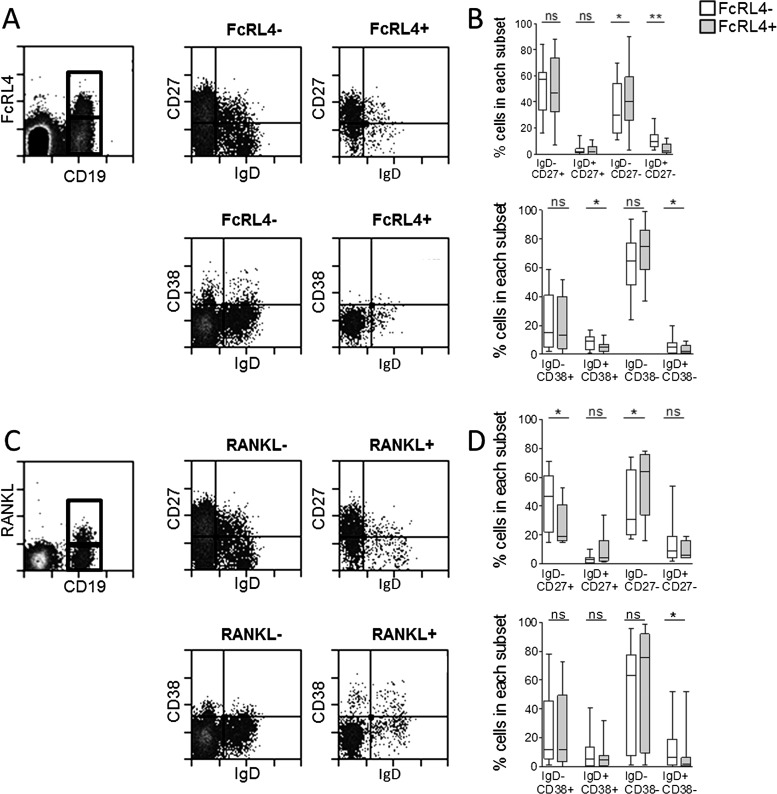
Memory phenoytpe of FcRL4+ and RANKL+ B cells in RA synovial fluid (SF). (A) Gating strategy for FcRL4+ and FcRL4− SF B cells and IgD/CD27 or IgD/CD38 expression. (B) FcRL4+ B cells and FcRL4− B cells are mainly switched memory IgD-CD27+ and IgD-CD27− and IgD-CD38− B cells. (C) Gating strategy for RANKL+ and RANKL− SF B cells and IgD/CD27 or IgD/CD38 expression. (D) RANKL+ B cells and RANKL− B cells are mainly switched memory IgD-CD27+ and IgD-CD27− and IgD-CD38− B cells. Wilcoxon matched paired test, *p<0.05, **p<0.01; n=10. Box length represents IQR; line represents median. *p<0.05, **p<0.01, Wilcoxon paired test. n=6–12.

**Figure 4:**
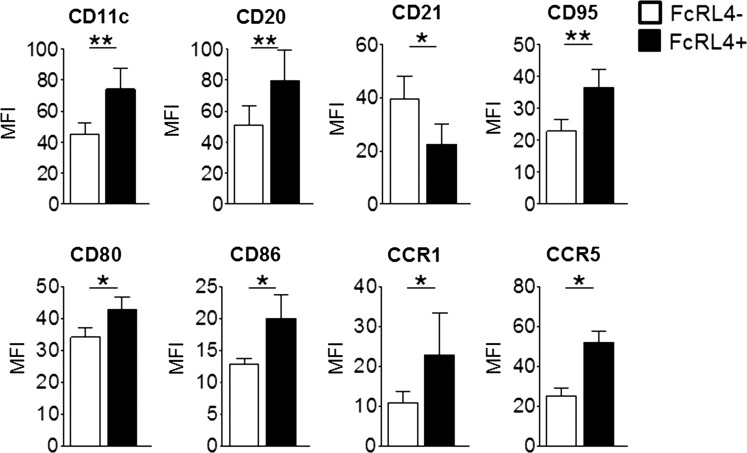
Phenotypic characterisation of FcRL4+ B cells in RA synovial fluid. FcRL4+ and FcRL4− B cells are phenotypically distinct. FcRL4+ B cells have higher expression of CD11c, CD20, CD95, CD80, CD86, CCR1 and CCR5, and lower expression of CD21 compared to FcRL4− B cells. *p<0.05, **p<0.01, Wilcoxon paired test; n=7–10.

The majority of the synovial fluid B cells, whether they expressed FcRL4 or not, displayed a memory B cell phenotype. FcRL4+ B cells were mainly IgD-CD27+ switched memory B cells (median 47%) or IgD-CD27- double-negative B cells (median 41%) (figure 3A). Of the FcRL4− B cell fraction, a median of 57% B cells were IgD-CD27+ and 30% were IgD-CD27-. There was a small but statistically significant enrichment of CD27-IgD- and CD27-IgD+ B cells in the FcRL4+ compared to the FcRL4− subset. Defined by the IgD/CD38 classification system, FcRL4+ B cells were predominantly IgD-CD38- (median 75%), which reflected the dominant phenotype of all synovial fluid B cells (figure 3A). RANKL+ B cells were also mainly IgD-CD27+ and IgD-CD38− switched memory B cells (figure 3B). Coexpression of FcRL4 and RANKL by each of the B cell subsets defined by IgD and CD27 expression is shown in online supplementary figure S1. Further assessment of cell surface markers highlighted several phenotypic differences that distinguish FcRL4+ and FcRL4− synovial fluid B cells. FcRL4+ B cells expressed higher levels of CD95, CD11c and CD20, and lower levels of CD21 in comparison with FcRL4− B cells (figure 4). FcRL4+ B cells showed elevated expression of the chemokine receptors CCR1 and CCR5. FcRL4+ B cells were also found to express significantly higher levels of the costimulatory molecules CD80 and CD86 compared with FcRL4− B cells.

### A pro-inflammatory role for FcRL4+ B cells in RA

**Figure 5:**
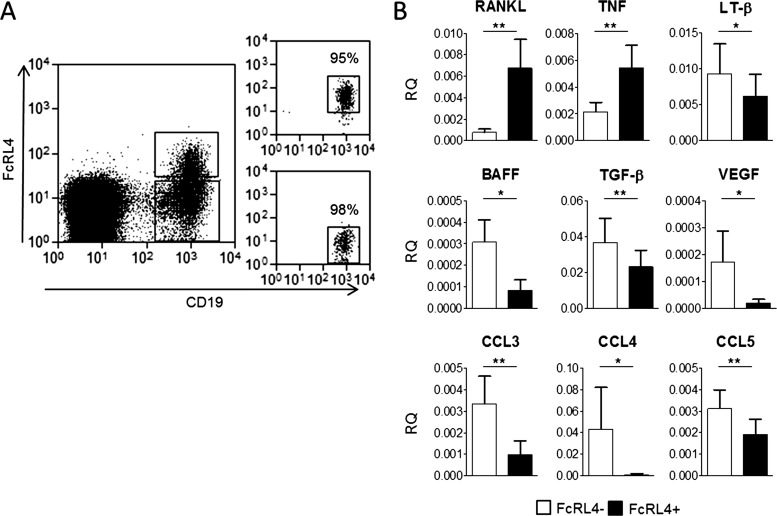
FcRL4+ B cells represent a pro-inflammatory population with a unique cytokine profile. (A) Gating strategy for FcRL4+ and FcRL4− B cell fractions sorted from RA synovial fluid using a MoFlo FACS sorter. Populations were sorted to a minimum purity of 95%. (B) Real-time PCR analysis of cytokine mRNA in FcRL4+ and FcRL4− B cells shows that FcRL4+ B cell express higher levels of RANKL and TNF-α than the FcRL4− B cell population. Conversely, FcRL4− B cells express higher levels of LT-β, B cell activating factor (BAFF), transforming growth factor-β (TGF-β), VEGF-α, CCL3, CCL4 and CCL5. *p<0.05, **p<0.01, Wilcoxon matched paired test; n=9.

Since FcRL4+ B cells were found to produce increased levels of RANKL, we sought to determine whether they exhibited an extended cytokine profile that was distinct from other B cells in the RA synovium. FcRL4+ and FcRL4− B cells were sorted from synovial fluid (as shown in figure 5A) and the expression of 43 cytokines was assayed using real-time PCR low-density arrays (see online supplementary figure S2 for full dataset). Comparison of FcRL4+ and FcRL4− B cells revealed that RANKL was expressed at significantly higher levels in the FcRL4+ B cell subset, confirming the observations made by flow cytometry at the protein level (figure 5B). Notably, FcRL4+ B cells expressed significantly higher levels of TNF-α mRNA than the FcRL4− B cell fraction (figure 5B). FcRL4+ and FcRL4− B cells also showed distinct expression patterns of several other cytokines. FcRL4− B cells showed significantly higher expression of lymphotoxin-β, B cell activating factor (BAFF), TGF-β, CCL3, CCL4 and CCL5 in comparison with FcRL4+ B cells (figure 5B).

## Discussion

This study identifies a pro-inflammatory B cell population in the inflamed synovium of patients with RA. This B cell subset is defined by its expression of FcRL4, and expresses significantly higher levels of mRNA for RANKL and TNF-α compared to other synovial B cells. The cytokine profile of the FcRL4+ B cell subset suggests that this population may be an important driver of chronic inflammation and bone destruction. TNF-α is recognised as a pivotal pro-inflammatory cytokine in inflammation and joint destruction in RA. Its pleiotropic actions include leukocyte and endothelial cell activation leading to pro-inflammatory cytokine production and leukocyte accumulation in the inflamed synovium, as well as stimulation of metalloproteinase release by fibroblasts and chondrocytes leading to cartilage and bone destruction.^15^ ^16^ RANKL and TNF-α are important mediators of osteoclast differentiation and activation. We therefore suggest that the B cell subset investigated here may contribute to bone erosion in patients with RA.

While FcRL4+ B cells are absent from the peripheral blood in healthy individuals, they have been detected in the blood of HIV-infected patients^17^ and malaria-exposed individuals^18^ where they have been found to exhibit a similar set of surface markers compared with that found in the tonsils and, as shown here, in the rheumatoid synovium. CD11c+CD21− B cells have been reported to accumulate in the peripheral blood of RA, systemic lupus erythematosus (SLE) and scleroderma patients^19^; these may represent a stage of B cell differentiation related to the FcRL4+ B cell population we describe here. However, the FcRL4+ subset is largely found in tissue, and can only rarely be identified in peripheral blood. It is conceivable that the CD11c+CD21− B cells may, if they enter the inflamed tissue, upregulate expression of FcRL4. The location of the FcRL4+ B cells outside the follicular clusters of B cells is reminiscent of a B cell subset described as interfollicular B cells. This latter group has been observed in the synovium, but beyond expression of activation-induced cytidine deaminase (AID), an enzyme involved in immunoglobulin gene hypermutation, their role in the synovium is not yet defined.^20^ The expression of CD80 and CD86 that we observed in FcRL4+ B cells is consistent with the activated phenotype described for tonsillar FcRL4+ B cells,^21^ and may point to a costimulatory role for FcRL4+ B cells in the synovium. FcRL4+ B cells were found to have elevated expression of CCR1 and CCR5 which are receptors for the chemokines CCL3, CCL4 and CCL5 (MIP-1α, MIP-1β and RANTES). Altered chemokine receptor expression may, therefore, account for the homing to and retention of FcRL4+ B cells in the inflamed synovium.

The unique expression profile of cytokines and chemokine receptors, together with the location of FcRL4+ B cells in synovial tissue, suggests FcRL4+ B cells are functionally distinct from other B cells present in the RA synovium. FcRL4+ B cells in tonsils do not express transcription factors necessary for plasmablast development^21^; however, the contribution of FcRL4+ B cells to local autoantibody production in the rheumatoid synovium remains to be investigated. Functional studies have described the FcRL4 receptor as capable of attenuating signalling through the B cell receptor, and also augmenting B cell activation through TLR9 signalling.^11^ ^12^ ^22^ TLR9 is an important driver of animal models of arthritis^23^ and putative TLR9 ligands such as DNA containing CpG motifs and single-stranded DNA have been found in RA synovial fluid.^24^ It is tempting to speculate that in RA FcRL4 may enhance immune activation through activation of TLR9 or, indeed, other TLRs which are known to be potently activated by disease-relevant ligands, such as immune complexes containing citrullinated proteins.^25^ While the functional contribution of the receptor in the RA synovium so far remains unknown, FcRL4 may serve as a useful marker for pro-inflammatory cytokine-producing B cells.

The effect of B cell targeting therapies such as rituximab cannot be entirely explained by the reduction of autoantibody levels.^26^ FcRL4+ B cells express significantly higher levels of CD20 compared with other synovial B cells, and we have previously shown that following treatment of RA patients with rituximab, levels of RANKL in the synovium are significantly diminished.^9^ Removal of the FcRL4+ subset of B cells from the inflamed synovium may, therefore, contribute to the anti-inflammatory and ant-ierosive effect of rituximab. Other mechanisms, such as costimulation inhibition and cytokine production blockade have been proposed but have not been thoroughly investigated. It is conceivable that removal of the cytokine and costimulatory signals produced by FcRL4+ B cells contributes to the clinical response seen in patients undergoing B cell depleting therapy. Current B cell depletion therapy for RA indiscriminately removes B cells and thus has the disadvantages of reducing patients’ responses to vaccination and can lead to a drop in total immunoglobulin levels. We propose that FcRL4 may represent a potential therapeutic target which would allow specific removal of a subset of pro-inflammatory B cells while leaving protective B cell immunity intact.

## Supplementary Material

Web supplement
