# Supplementary figures and images for: Expression of FcRL4 defines a pro-inflammatory, RANKL-producing B cell subset in rheumatoid arthritis
Source: Ann Rheum Dis. 2014 Jan 15;74(5):928–35. doi: 10.1136/annrheumdis-2013-204116 (PMC4392201; doi:10.1136/annrheumdis-2013-204116)

Supplementary Figure S1

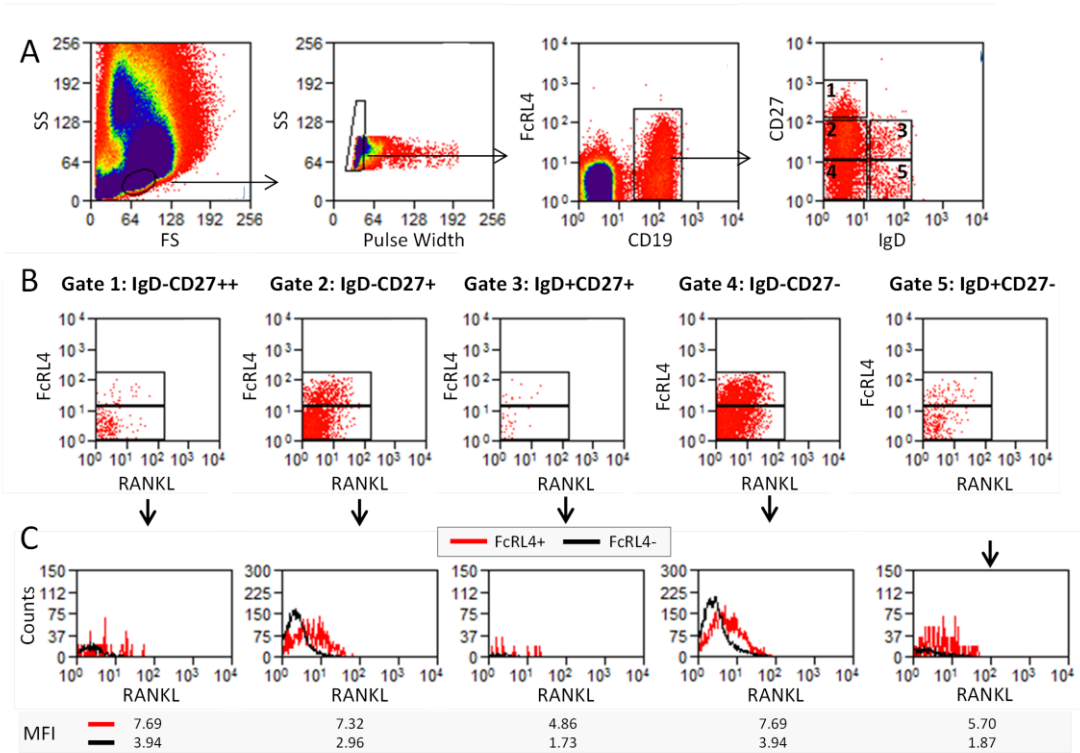

Supplementary Figure S2

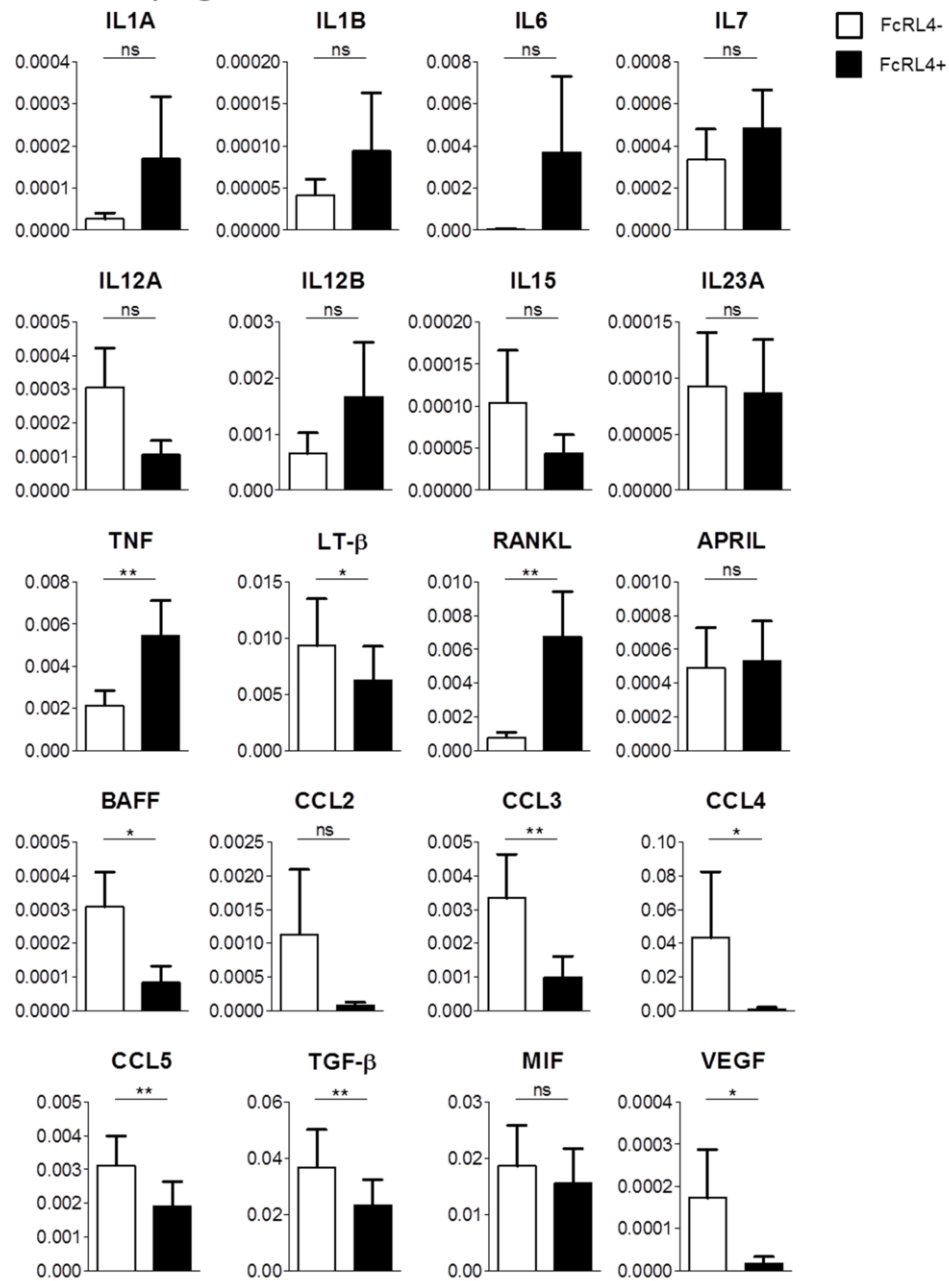

Supplement: Web supplement [file annrheumdis-2013-204116-s1.pdf]
